# Satisfaction with focused antenatal care service and associated factors among pregnant women attending focused antenatal care at health centers in Jimma town, Jimma zone, South West Ethiopia; a facility based cross-sectional study triangulated with qualitative study

**DOI:** 10.1186/1756-0500-7-164

**Published:** 2014-03-19

**Authors:** Fantaye Chemir, Fessahaye Alemseged, Desta Workneh

**Affiliations:** 1 Department of Midwifery and Nursing, Jimma University, Jimma, Ethiopia; 2 Department of Epidemiology and Biostatistics, Jimma University, Jimma, Ethiopia

**Keywords:** Focused antenatal care, Satisfaction, Dissatisfaction, Component wise satisfaction

## Abstract

**Background:**

Client satisfaction is essential for further improvement of quality of focused antenatal care and to provide uniform health care services for pregnant women. However, studies on level of client satisfaction with focused antenatal care and associated factors are lacking. So, the purpose of this study is to assess satisfaction with focused antenatal care service and associated factors among pregnant women attending focused antenatal care at health centers in Jimma town.

**Methods:**

A facility based cross-sectional study involving both qualitative and quantitative methods of data collection was used from Feb 1-30/2013. Three hundred eighty nine pregnant women those come to the health centers were included in the study. A semi-structured questionnaire and focus group discussion guide was employed to obtain the necessary information for this study. Quantitative data was analysed using SPSS for windows version 16.0. Logistic regression model was used to compare level of satisfaction by predictors’ variables. Qualitative data was analyzed based on thematic frameworks to support the quantitative results.

**Result:**

More than half of the respondents (60.4%) were satisfied with the service that they received. As to specific components, most of the respondents (80.7%) were satisfied with interpersonal aspects, and 62.2% were satisfied with organization of health care aspect. Meanwhile, 49.9% of the respondents were not satisfied with technical quality aspect and 67.1% were not satisfied with physical environment aspect. Multivariate logistic regression analysis result showed that type of health center, educational status of mother, monthly income of the family, type of pregnancy and history of stillbirth were the predictors of the level of satisfaction. The study found out that dissatisfaction was high in mothers utilizing service at Jimma health center, in mothers with tertiary educational level, in mothers with average monthly family income >1000birr, in mothers with unplanned pregnancy and in mothers with history of stillbirth.

**Conclusions:**

Even though greater percentages of women (60.4%) were satisfied with the focused antenatal care service, the level of satisfaction was lower compared to other studies. The investigator recommends that patient feedback should be recognized as a legitimate method of evaluating health services in the health center as a whole.

## Background

Even though between 1990 and 2010, maternal mortality worldwide dropped by almost 50%, every day, approximately 800 women die from preventable causes related to pregnancy and childbirth and 99 per cent of all maternal deaths occur in developing countries [1]. Sub-Saharan Africa had the highest maternal mortality rate (MMR) at 500 maternal deaths per 100 000 live births. Ethiopia is one of the few countries that account for most of the maternal deaths; others include India, Nigeria, Democratic Republic of the Congo, Pakistan, Sudan, and Indonesia [2]. According to 2011 Ethiopian Demographic Health Survey(EDHS), the maternal mortality rate of Ethiopia is 676/100,000 live births [3]. In Ethiopia, like in many developing countries, the causes of maternal deaths are mainly attributed to the three delays; that is delay in seeking care, delay in reaching appropriate care and delay in receiving care. Delay in receiving care can happen due to inadequate skilled personnel in emergency obstetric care, inadequate supplies and equipment and poor quality of services [4].

The government of Ethiopia is committed to achieving millennium development goals (MDG) 5, to improve maternal health, with a target of reducing the maternal mortality rate (MMR) by three-quarters over the period 1990 to 2015. Accordingly, the Federal Ministry of Health (FMoH) of Ethiopia has applied multi-pronged approaches to reducing maternal and newborn morbidity and mortality. Improving access to and strengthening facility-based maternal and newborn services is one such approach, and is also a health sector development plan (HSDP) strategic objective. The Antenatal Care is considered as one of a focused strategy to reduce maternal mortality. According to 2011 Ethiopian Demographic Health Survey (EDHS) report, nationally thirty-four percent of pregnant mothers who gave birth in the five years preceding the survey received antenatal care from a skilled provider for their most recent birth and regional (Oromia) prevalence is 31%. Few developing countries including Ethiopia have fully embraced and implemented the focused antenatal care (FANC) model. Even in countries adopting it as their antenatal care (ANC) programme, it is not fully implemented due to lack of personnel and structural changes [5-8].

One of the important problems which are continuously faced these days is the lack of good quality antenatal care and gaining client satisfaction, which are of important responsibilities of the higher authorities and staffs in the health care system [9]. Evaluating to what extent patients are satisfied with health services is clinically relevant, as satisfied patients are more likely to comply with treatment, take an active role in their own care, to continue using medical care services and recommend center‘s services to others [10]. A satisfied patient will recommend center‘s services expressing their satisfaction to four or five peoples, while a dissatisfied patient on the other hand will complain to twenty or more [11]. It is also essential to identify the factors involved in dissatisfaction if a good health care system is sought [12].

Despite the fact that client satisfaction is essential for further improvement of quality of focused antenatal care and to provide uniform health care services for pregnant women, little is known about the levels and associated factors of satisfaction in Ethiopia in general and no data in the study area in particular. Therefore, this paper aims to have certain contribution in closing this gap.

## Results

A total of 389 pregnant women were enrolled in the study giving a completion rate of 100%; of which 142(36.5%) from Mendera Kochi health center, 136(35%) from Higher 2 health center and 111(28.5%) from Jimma health center.

### Socio-demographic characteristics

The largest numbers of pregnant women belong to the age range between 20 to 29 years 284(73%) followed by age range 30–39 years 73(18.8%) with mean age of 25 years. Three hundred seventy six (96.7%) of the women were married and the rest were single. Regarding ethnic and religious distribution of respondents, the predominant ethnicities were Oromo 241(62%) followed by Guragae 48(12.3%) while the dominant religion was Muslim 236(60.7%) succeeded by orthodox 112(28.8%). With regard to occupation, two hundred sixty four (67.9%) of pregnant women were housewives followed by merchants and employees accounting for 46(11.8%) and 40(10.2%) respectively. One hundred forty six (37.5%) of the women attended primary education (grade 1–8) succeeded by those who had no education 111(28.5%). The greatest number of the respondents, 128(32.9%) had average family monthly income below 500birr (Table 1).

**Table 1:** Socio-demographic and economic factors of pregnant women attending FANC at health centers in Jimma town, Feb. 2013

**Variable**		**Number (N = 389)**	**Percent**
Age(in years)	15-19	31	8.0
	20-29	284	73.0
	30-39	73	18.8
	40-49	1	0.2
Ethnicity	Oromo	241	62.0
	Guragae	48	12.3
	Kefa	33	8.5
	Yem	23	5.9
	Amhara	21	5.4
	Others	23	5.9
Occupation	House wife	264	67.9
	Merchant	46	11.8
	Employee*	40	10.2
	Daily laborer	15	3.9
	Farmer	12	3.1
	Others	12	3.1
Educational status	No formal education	111	28.5
	Primary	146	37.5
	Secondary	87	22.4
	Tertiary	45	11.6
Marital status	Married	376	96.7
	Single	13	3.3
Religion	Muslim	236	60.7
	Orthodox	112	28.8
	Protestant	39	10.0
	Jova	2	0.5
Average family monthly income(in birr)	<500	128	32.9
	501-750	57	14.7
	751-1000	87	22.4
	>1000	117	30.0

*Employee includes governmental, non-governmental and private.

### Obstetric and reproductive health profile

Among the total studied clients, 283(72.8%) of them have given birth for one to four children followed by those who have never given birth and who gave birth for five or more children accounting for 81(20.8%) and 25(6.4%) respectively. Among who have given birth 307(78.9%), 34(11.04%) of them end up with stillbirths. Seventy one (18.3%) of the women had previous history of abortions. From women who had previous history of pregnancy, 185(60.2%) had at least one history of antenatal care. The majority of the respondents’ 320(82.3%) agreed that their current pregnancy is planned and wanted (Table 2).

**Table 2:** Obstetric profiles of pregnant women attending FANC at health centers in Jimma town, Feb. 2013

**Variable**		**Number**	**Percent**
Parity (N = 389)	Nulliparous	81	20.80
	Multipara	283	72.80
	Grand multipara	25	6.40
Type of pregnancy (N = 389)	Planned	320	82.30
	Unplanned	69	17.70
History of abortion (N = 389)	Had	71	18.30
	No	318	81.70
History of still birth (N = 307)	had	34	11.04
	No	273	88.96
History of FANC (N = 307)	Yes	184	60.06
	No	123	39.94
Current visit (N = 389)	First	116	29.80
	Second	144	37.00
	Third	87	22.40
	Fourth or more	42	10.80

### Current health condition and knowledge of pregnant women about importance of fanc

From the total study clients, 289(74.3%) of them responded that their current health condition is ‘good’ , while the rest 100(25.7%) is ‘poor’. Two hundred ninety four (75.6%) of the clients had ‘good knowledge’ regarding importance and objectives of focused antenatal care.

### Women’s satisfaction with fanc services

**Figure 1:**
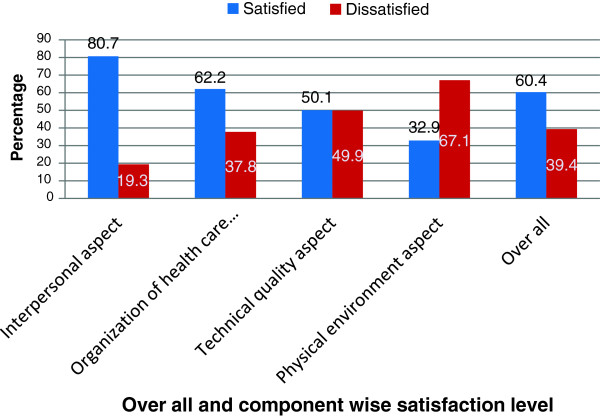
Level of satisfaction with FANC services among pregnant women attending FANC at health centers in Jimma town, Feb. 2013.

In overall, 235(60.4%) of the women were satisfied and the rest 39.4% were dissatisfied with the focused antenatal care (FANC) services (Figure 1). Based on component wise level of satisfaction, greater proportion of satisfaction was recorded on interpersonal skill aspect followed by organization of health care aspect which accounts for 314(80.7%) and 242(62.2%) respectively (Figure 1). The finding was supported by qualitative results as: majority of the discussant from the three HCs share the idea that ……..*they were very happy that the service providers have much respect for them and they are eager to help them. One of the discussant from mendera kochi HC also said that “……..I was happy because they tried to ask me what I feel and they had given me information related to pregnancy”.* Greater proportion of dissatisfaction was recorded on physical environment part followed by dissatisfaction with technical quality which accounts for 261(67.1%) and 194(49.9%) respectively (Figure 1). The findings on areas of dissatisfaction was supported by qualitative findings as: many of the discussants forwarded that: *……..they are not happy and not sure on the appropriateness of physical examinations done on them because they do have questions on the technical quality of students since in the majority of cases the service was rendered by students (Jimma HC and Higher two HC).* One of the discussant from Higher 2 HC said that *“…….I was not happy on the examinations they had performed; they didn’t take even a minute to carry out and they look lacking confident”.*

### Perceived causes of dissatisfaction and client suggestions to make fanc services more satisfying to pregnant women

In this study, clients were asked for any comments and suggestions regarding the FANC service they have received. The perceived cause of dissatisfaction forwarded by the majority were long waiting time 127(32.6%), overcrowding in the clinic during morning time 101(26%) and poor laboratory services 98(25.2%). Possible suggestions raised by most of the clients include; minimize waiting time 128(32.9%), prepare appropriate waiting room 96(24.7%), and improve ventilation system of the office and waiting space 82(21.1%) (Table 3).

**Table 3:** Perceived cause of dissatisfaction and suggestions among pregnant women attending FANC at health centers in Jimma town, Feb. 2013

***# Perceived cause***	**Frequency**	**Percentage**
Poor laboratory service	98	25.2
Crowding in the clinic in the morning	101	26
No listening complaints of pregnant women	56	14.4
Long waiting time	127	32.6
Absence of sonar test	46	11.8
Unfavourable waiting area	17	4.3
Others	17	4.3
***# Client suggestions***		
Avoid talking each other too much and give more attention to the client	46	11.8
Give information related to pregnancy while waiting time	52	13.4
Improve ventilation system of the office and waiting space	82	21.1
Prepare appropriate waiting room	96	24.7
Minimize waiting time	128	32.9
Increase waiting space	48	12.3
Improve behavior of supporting staff	33	8.5
Others	24	6.2

**#**More than one answer was possible.

### Factors associated with satisfaction

Both bivariate and multivariate binary logistic regression analysis were made to identify predictors of satisfaction. The bivariate analysis result revealed that health center type, socio-demographic variables such as ethnicity, occupation, educational status, religion, and family monthly income of the mother, obstetric profile variables such as parity, type of pregnancy, focused antenatal care (ANC) history, and number of visit and client knowledge about importance of focused antenatal care (FANC) were significantly associated with satisfaction with focused antenatal care services. The above mentioned significant variables and those with p-value less than 0.25 in the crude analysis (marital status and history of stillbirth) were again entered in to multivariate logistic model to control for confounding. The only variables with p-value >0.25 in crude analysis and also excluded from multivariate logistic analysis were age, abortion history and health condition. The variables with p-value less than 0.05 in multivariate analysis were taken as significant predictors of satisfaction and the rest were refuted. Variables which significantly predict level of satisfaction with focused antenatal care services include: type of health center, socio-demographic variables: average monthly income of family and educational status of the mother, and obstetric profile variables: type of pregnancy and history of stillbirth. It was observed that pregnant women who were satisfied with FANC services were likely to be: those who utilize service in Mendera Kochi (AOR = 4.93, 95% CI: 1.82, 9.74) and Higher Two HCs (AOR = 4.91, 95% CI: 1.44, 16.73), those who have no formal education (AOR = 32.63, 95% CI: 3.55, 299.93) and have attended primary education (AOR = 16.87, 95% CI: 2.08, 137.18), those with average family monthly income below 500birr (AOR = 8.84, 95% CI: 2.77, 28.19) and above 750 and below 1000birr (AOR = 3.03, 95% CI: 1.19, 7.72), those with planned pregnancy (AOR = 5.05, 95% CI: 1.86, 13.66) and no history of stillbirth (AOR = 5.47, 95% CI: 1.64, 18.25) as compared to their respective referents(Table 4).

**Table 4:** Predicators of satisfaction on multivariate analysis among pregnant women attending FANC at health centers in Jimma town February, 2013

**Variable**		**Satisfied N (%)**	**AOR(95% CI)**
Health center	Mendera Kochi	96(67.6%)	4.93(1.82, 9.74)*
	Higher two	109(80.1%)	4.91(1.44, 16.73)*
	Higher one (Jimma)	30(27.0%)	1
Average family monthly income(in birr)	<500	108(84.4%)	8.84(2.77, 28.19)*
	501-750	35(61.4%)	1.59(0.49, 5.17)**
	751-1000	52(59.8%)	3.03(1.19, 7.72)*
	>1000	7(30.4%)	1
Type of pregnancy	Planned	203(63.4%)	5.05(1.86, 13.66)*
	Unplanned	32(46.4%)	1
History of still birth	Yes	17(50.0%)	1
	No	178(65.2%)	5.47(1.64, 18.25)*

**Note:** *statistically significant associations in multivariate analysis. ** P value of greater than 0.05 in multivariate analysis‘1’ reference group.

## Discussion

According to this study it was found out that overall satisfaction with focused antenatal care service in the study population was 60.4%. This is somewhat similar with findings of a study conducted in Malaysia (56.7%) but lower than other studies conducted in Addis Ababa, Ethiopia (89.2%), Sweden (82%) and Thailand (71.8%) [13,20-22]. The difference could be due to subjective nature of the subject matter; because measure of satisfaction needs standardized scales and tools for accurate measurement but most of the literatures measure satisfaction with simple yes/no response category. And also could be attributed to study period difference due to the increase in expectation of patients to the service they are going to receive with rapid advancement in technology and peoples thinking and lifestyle and also the study is conducted in urban setting only. Even if greater percentage of overall satisfaction was reported in different literatures, there is a difference in satisfaction level in different aspects of focused antenatal care services. The results of this study indicated that more than three fourth (80.7%) and over half (62.2%) of pregnant women were satisfied with interpersonal and organization of health care aspects of focused antenatal care services respectively. However, half (49.9%) and 67.1% of pregnant women were dissatisfied with technical quality and physical environment aspects of focused antenatal care services respectively. The finding shares some inconsistencies in terms of higher level of satisfaction with technical quality aspect and lower level of satisfaction with organization of health care aspect with other studies conducted in Malaysia and Thailand [13,21]. The reason for the inconsistencies might be attributed to difference in cultural setting in providers and receivers and due to differences in set up in which the studies are conducted at hospital antenatal units.

The perceived cause of dissatisfaction forwarded by the majority were long waiting time (32.6%), overcrowding in the clinic during morning time (26%) and poor laboratory services (25.2%). Possible suggestions raised by most of the clients include minimize waiting time (32.9%), prepare appropriate waiting room (24.7%), and improve ventilation system of the office and waiting space (21.1%). This finding was supported by other studies conducted in Bangladesh and Oman [23,24].

This study found that pregnant women who were utilizing service at Higher 2 HC (80.1%) were more satisfied than Mendera kochi health center (67.6%) and Jimma HC (27%). The lower satisfaction level of pregnant women attending focused antenatal care at Jimma health center might be associated with high client flow per day as compared to other HCs and preferable by majority of the people residing on the center of the town due to its favorability in terms of geographic location. While the other health centers more suitable for mothers from rural settings. It was also observed that pregnant women with low level of education were more likely to be satisfied with FANC services than those who had higher level of education. The possible reason why women with higher level of education were dissatisfied was because women with a higher level of education are probably more vocal and information-seeking and know what to expect. This finding agrees with findings of a study in Malaysia [13,20]. However, it disagrees with a study done in Sweden in which patients with a low level of education were less likely to be satisfied than women with a high level of education. The reason forwarded for lower level of satisfaction among mothers with low educational level was because there could be a discrepancy in communication between women with a low level of education and the service providers; it is known that communication is facilitated by similar social and educational background. The likelihood of satisfaction by antenatal care service was lesser among women whose monthly income was 1000 birr or more or the higher monthly income the lower the satisfaction and vice versa which agreed with study conducted at Addis Ababa Ethiopia. The poor economic condition and living below the poverty line with low monthly income of respondents could have made them unable to deal with modern medical services or exposure to other kind of facilities. This made patients satisfied with any services that they were provided [22]. The chance of satisfaction on the focused antenatal care service rendered was lower to a statistically significant level among women who had unplanned pregnancy and history of stillbirth which agrees with findings of a study conducted at Addis Ababa Ethiopia by Workneh. Women who had unplanned pregnancy may be too sensitive in terms of privacy and confidentiality due to possible stigma if the pregnancy is out of the wedlock. And also women who had unplanned pregnancy experience greater relationship instability than women whose pregnancies were intended. Lack of faith on the service they were receiving and associating cause of stillbirth with constraints from the service they received in their past pregnancy focused antenatal care follow up may be a reason for decreased likelihood of satisfaction among pregnant women with previous history of stillbirths [22,25].

### Strength and limitations

The study employed standardized five point likert scale and reliable tools for measurement of satisfaction. The study also involved both quantitative and qualitative methods of data collections to maximize the reliability of the data collected.

Social desirability bias could have affected the quality of data collected because study subjects might get difficulty to answer dissatisfaction in the presence of an interviewer. However, interview was conducted in a separate room by non-staff members to minimize the bias.

## Conclusions

Even though greater percentages of women (60.4%) were satisfied with the focused antenatal care service, the level of satisfaction was lower compared to other studies. Also the clients were dissatisfied with physical environments of the health center and technical quality of the providers. Since, patient satisfaction is an increasingly important issue both in evaluation and shaping of health care, it should be carried out routinely in all aspects of health care to improve the quality of health services. Long waiting time, overcrowding in the clinic during morning time, and poor laboratory services were some of the constraints perceived by majority of pregnant women as a cause of dissatisfaction. Health center admistrators should increase the staff strength to cope with the client load, to increase the consultation time and decrease overcrowding. The waiting time in reception area before getting focused antenatal service should be decreased by better use of resources and by increasing man power.

## Methods

### Study area and period

The study was conducted at Jimma town health centers from February 1-30/2013. Jimma town is located 357 kms South West of Addis Ababa with a total projected population of 151,010. The town has 3 health centers. Total estimated number of pregnant women in each health center encompasses 1610 for Jimma Health Center, 1960 for Higher 2 Health center and 2044 for Mendera Kochi Health center respectively. Average number of client flow per day is 9 for Higher 2 Health center, 12 for Jimma Health center and 6 for Mendera Kochi Health center.

### Study design

A facility based cross-sectional study design with both quantitative and qualitative methods of data collection was employed.

### Source and study population

All pregnant women utilizing focused antenatal care (FANC) services in the health centers were the source populations. And pregnant women utilizing focused antenatal care service in the health centers during data collection period were considered as study population.

### Sampling procedure

The sample size for quantitative study was determined by the single population proportion formula by considering 56.7% proportion of satisfaction of a study done in Malaysia [13] with a marginal error of 5% between the sample and the population at 95% confidence level, which were 389. Pregnant women who were registered for antenatal care during data collection period were taken until the required sample size was fulfilled after proportionally allocating the sample population to each health centers. For qualitative study six FGDs; two in each health center was conducted. A total of 48 pregnant women with 7–10 pregnant women in each FGD were participated. A convenience sampling technique was used to select pregnant women for the FGD by taking health centre as homogeneity criteria.

### Measurement

The data were collected using pre tested semi-structured questionnaires and measurement scales that were adapted from the review of literatures [13-17]. The questionnaire contains 49 items which were related to socio-demographic characteristics, obstetric profile; current health condition and knowledge of pregnant women on purpose and objectives of focused antenatal care (FANC) and measure of satisfaction. The outcome variable satisfaction was assessed using a 5-point Likert scales ranging from dissatisfied to fully satisfied (1 to 5 points) with a 27 items. The satisfaction scale had the internal consistency or reliability score ranging from 0.73 to 0.95(Table 5).

**Table 5:** reliability score for measure of satisfaction scale

**Scale**	**Standardized Cronbach’s alpha**
Overall satisfaction	.95
Art of care	.91
Technical quality	.81
Physical environment	.78
Organization of health care	.73

Focus group interview guideline was used to guide and probe the focus group discussion. The focus group interview guideline includes probing questions on areas of care clients satisfied, areas of care clients not satisfied and client suggestions regarding how to make services more satisfactory.

Pretesting of the questionnaire was conducted among 5% of pregnant women other than the study population in Serbo health center focused antenatal care (FANC) unit prior to actual data collection to assess the face validity of the questionnaire.

### Data collection

A total of 3 data collectors and 3 supervisors were recruited for data collection procedure. The data collectors and supervisors were non-staff members and female in gender to make clients feel more confidential and anonymous. The questionnaires were filled by direct face to face semi-structured interview. Clients were interviewed at exit. The clients were interviewed outside the service room far away from employees and the data collectors were non-staff personnel’s to assure confidence and anonymity. The data collectors and supervisors were trained a week ahead of the actual data collection period on data collection process to standardize interviews and reduce interviewer biases. For qualitative study; clients were asked to express their satisfaction following the FGD guideline. All discussions were tape recorded & field notes were taken and transcribed to texts immediately. Each discussion took one to two hours.

### Data analysis

Following the data collection, data were coded, and entered to a computer using Epidata version 3.1 and then exported to SPSS version 16.0 for analysis. Descriptive statistics and binary logistic regressions analysis were performed. In the binary logistic regression, both binary and multivariate analyses were carried out. All the variables were entered in to bivariate analysis and those explanatory variables with a p value < 0.25 in crude analysis was considered as a candidate for multivariate analysis and those variables with a p value < 0.05 in multivariate analysis was considered as significant predictor of satisfaction. Finally, the result of the analysis was presented in texts, tables and graphs as appropriate. For qualitative study, first individual, pre-labeled tapes were transcribed and then translation and back translation of the transcription was performed. The discussion was conducted in Amharic and Oromiffa so that it was translated to English and back translated to Amharic and Oromiffa. Next completed transcription was compared with hand written notes to fill inaudible phases or gaps in tapes. The data were color-coded and grouped based on thematic frameworks (three thematic areas). Concepts were extracted from themes and presented in narratives & used to support the quantitative results.

### Operational definitions

Cut off point for client satisfaction: − Since each item had 5 point Likert Scale which ranges between 1 and 5; the scores for each domain was calculated by summing the answers to all items in each domain. Clients’ overall and component wise level of satisfaction was classified into two categories satisfied and dissatisfied by using cut of point calculated using the demarcation threshold formula: {(total highest score-total lowest score)/2} + Total lowest score [13,18].

#### Knowledge

One point was given for the correct answers and zero for the incorrect answers. The knowledge scores were divided to two levels which are good knowledge and poor knowledge using the mean knowledge score as the cutoff point [15,19].

#### Current health condition

For mothers that didn’t have any complain during current visit, their health conditions were considered as good. For the mothers with any complain including aggravations of minor disorders of pregnancy, their current health conditions were considered as not good.

### Ethical considerations

Before the data collection, ethical clearance letter was obtained from the ethical committee of the college of public health and medical sciences, Jimma University. A formal letter of cooperation from the health centers was granted prior to data collection. Introduction of the study, method of the questioning and confidentiality letter was attached to cover page of the questionnaires. The respondents were informed about the purpose of the study, & their oral and written consent was obtained. The respondents’ right to refuse or withdraw from filling out the questionnaire was fully maintained. The information provided by each respondent was kept strictly confidential.

## Abbreviations

ANC: Antenatal care; ASQ: Antenatal satisfaction questionaire; EDHS: Ethiopian demographic health survey; FANC: Focused antenatal care; FGD: Focus group discussion; FMoH: Federal ministry of Health; HC: Health center; HSDP: Health sector development plan; MDG: Mellinium development goals; MMR: Maternal mortality ratio.

## Competing interests

The authors declare that they have no competing interests.

## Authors’ contribution

FC involved in designing of the study, data collection, data analysis, drafting and critically reviewing the manuscript. Likewise, FA and DW involved in designing of the study, analysis of the data and critically reviewing the manuscript. All authors read and approved the final manuscript.

## Authors’ details

FC is lecturer of maternal health nursing in college of public health and medical science of Jimma University, and FA is associate professor of epidemiology in college of public health and medicinal science of Jimma University. DW is lecturer of maternal health nursing in the college of public health and medical science of Jimma University. All authors are currently staff members in their respective department in Jimma University.
